# Basic physical properties and potential application of graphene oxide fibers synthesized from rice husk

**DOI:** 10.1038/s41598-023-45251-8

**Published:** 2023-10-20

**Authors:** J. R. Castro-Ladino, C. A. Cuy-Hoyos, J. J. Prías-Barragán

**Affiliations:** 1 Grupo de Investigación en Tecnologías Emergentes (GITEM), Universidad de los Llanos, Villavicencio, 500001 Colombia; 2 Interdisciplinary Institute of Sciences, Doctoral Program in Physical Sciences and Electronic Instrumentation Technology Program at Universidad del Quindío, Armenia, 630004 Colombia

**Keywords:** Materials science, Electronic devices

## Abstract

The synthesis method and correlation between compositional, vibrational, and electrical properties in graphene oxide fibers (GOF) are presented and discussed here, as well as a potential application through the development of a heater device based on GOF. The GOF samples were synthesized from rice husk (RH), via a thermal decomposition method, employing an automated pyrolysis system with a controlled nitrogen atmosphere, varying carbonization temperature (T_CA_) from 773 to 1273 K. The compositional analysis shows peaks in the XPS spectrum associated with C1s and O1s, with presence of hydroxyl and epoxy bridges; the oxide concentration (OC) of samples varied from 0.21 to 0.28, influenced by T_CA_. The GOF samples exhibit fiber morphology, vibrational characteristics which are typical of graphene oxide multilayers, and electrical behavior that scales with OC. The electrical response shows that OC decreases and increases electrical conductivity at the polycrystalline phase, possibly attributed to the desorption of some oxides and organic compounds. In addition, physical correlations between OC and its vibrational response showed that decreasing OC increases edge defect density and decreases crystal size as a result of thermal decomposition processes. The correlation between OC and physical properties suggests that by controlling the OC in GOF, it was possible to modify vibrational and electrical properties of great interest in fabrication of advanced electronics; consequently, we show a potential application of GOF samples by developing an electrically controlled heater device.

## Introduction

Graphene materials have aroused great interest in basic and applied research^1–3^. However, these high-quality materials have a considerable cost and low availability. Precursor material from biomass, such as RH is an alternative low-cost, highly available, green method to produce carbon materials^4–7^, like GOF. RH is an abundant agro-industrial waste product, which can generate negative impacts on the environment, as well as on human and animal health; however, it has interesting compositional characteristics, as reported by Teo E.Y.L. et al.^5^, among others^8–10^. This aspect promotes RH as an alternative precursor in the production of graphite oxide materials^1,4^. Also, these materials are important in applications, such as green nanocomposites^8^, supercapacitors^11–14^, conductive materials^15^, adsorbent materials^1,16^, nanomaterials in biomedical applications^17^, batteries^18,19^, electronic devices^20,21^, heater devices^4^, solar cells^22^, and sensors^14,23,24^, among others.

Numerous methods have been developed to synthesize GO, including Hummers’ and its variants, Brodie’s Staudenmaier’s, among others^25,26^. However, these methods are complex and involve using toxic materials and solutions that harm the environment. Recently, biomass materials have been used to prepare materials based on graphene by employing the thermal decomposition method^25,27^. Also, first-stage thermal decomposition (FSTD) has been employed to synthesize GO from biomass precursors, like bamboo and RH^4,28^.

Several works have synthesized GO and reduced graphene oxide (rGO) using traditional methods^26^ and recently, it was synthetized via a FSTD method, employing RH biomass as source material^8,27,29^; furthermore, their morphological, compositional, vibrational, electrical, and physicochemical properties have been studied^4,8,30–33^ and the effect of T_CA_ on these physical properties has been reported^21,34,35^, in addition to their possible technological applications^4,36–38^. Moreover, some studies revealed that physical properties in GO can be influenced by tuning oxide concentrations^39–43^. Then, different experiments were carried out to measure the electrical response of GO via I–V curves method^43,44^, showing that the temperature level of the medium in which the experiment is performed and the reduction or synthesis temperature influences electrical conductivity and the OC^39,40,43^. The vibrational characteristics were analyzed via Raman; the Raman spectrum of GO shows general features around 1360, 1600, and $2700\, {cm}^{-1}$, which correlate with D, G, and 2D bands, respectively^4,8,29,40^. The OC variation generates significant changes in the GO Raman spectrum, such as the shift and intensity variation of the D and G bands, as a result of the modification of the functional groups, as well as the 2D and D + G bands and variation in the $\frac{{I}_{D}}{{I}_{G}}$ ratio^39,42^. Additionally, in the FTIR spectra, it has been reported that changes in the position and intensities of the peaks in GO samples can be attributed to the different levels of OC^40,45^. Compositional characteristics have been studied, employing XPS and two main peaks at 284 and $531\, eV$ were observed and attributed to C1s and O1s, respectively. As the OC increases, the intensity of the C–C peak due to the sp^2^ carbon bond in graphite gradually decreases, and increases the intensity of peaks associated to functional groups, such as hydroxyl, carboxyl, and epoxy groups^33,39,42^.

However, although these physical properties have been studied in GO, the correlation amongst these physical properties in GOF remains unknown, and lacks a deep physical interpretation. Therefore, this work presents the correlation among the compositional, vibrational, and electrical properties, and the effect of the OC, crystal size, and defects density on the electrical conductivity of GOF samples. Also, the synthesis procedure is presented in GOF samples obtained from the RH by using the FSTD method, the OC was estimated through XPS analysis, and the vibrational characteristics were measured via Raman spectroscopy.

## Results and discussion

### Oxide concentrations

The OC was quantitatively determined by Eq. (1), deduced from the method presented by Carvalho et al. in^46^, based on the peak-to-peak binding energy difference of the high-resolution XPS C1s spectrum.

1 $$OC={\left(\frac{\Delta {E}_{C1S}-{E}_{0}}{A}\right)}^\frac{1}{2}+{C}_{l}.$$

In Eq. (1), OC is oxide concentration; $\Delta {E}_{C1S}$ is the binding energy (BE) shift between the C sp^2^ peak and functional group C─OH (hydroxyl) peak; ${E}_{0}$ is the fundamental state of energy or energies imposed by the presence of carbon atoms; $A$ is a parameter obtained from the fit and related to the energy value of $52.3 \,eV$, it can be associated to a slope; and ${C}_{l}$ is independent of the energies and depends on a concentration due to the influence of the nuclei with a value of $0.122$^46^.

**Figure 1:**
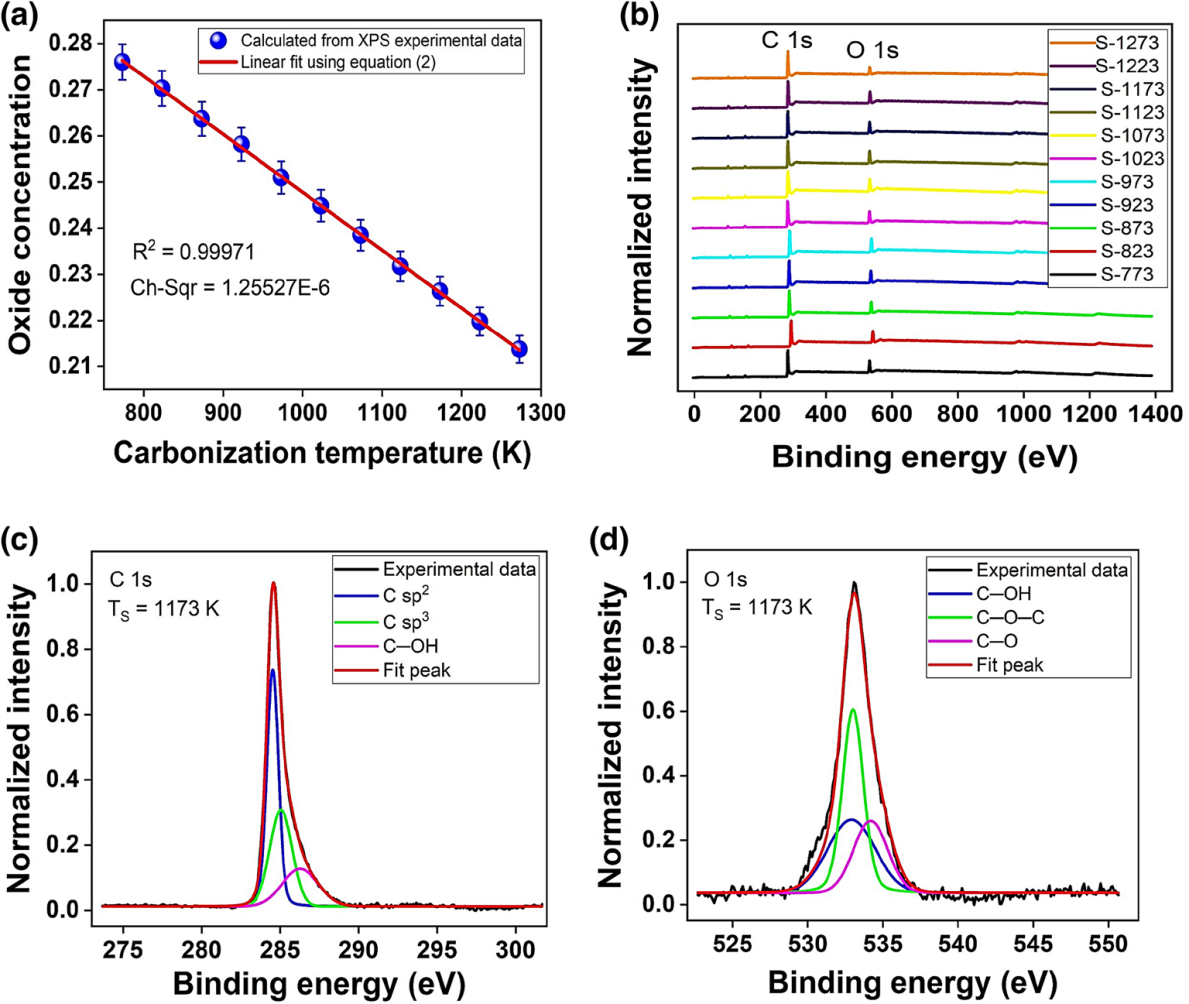
(**a**) Carbonization temperature influence in OC of GOF samples obtained by varying T_CA_ from 773 to 1273 K, (**b**) XPS spectra of GOF samples synthesized to T_CA_ from 773 to 1273 K, (**c**) High-resolution C1s spectra, and (**d**) High-resolution O1s spectra of S-1173.

Figure 1a shows OC as a function of T_CA_; as the synthesis temperature decreases, the OC increases from 0.21 (T_CA_ = 1273 K) to 0.28 (T_CA_ = 773 K). The fit corresponds to a linear relationship given by Eq. (2) and this $OC=f\left({T}_{CA}\right)$ can be attributed to the oxides and organic compounds desorption. The negative slope probably indicates that the higher the synthesis temperature is, the more it promotes oxide and organic compounds desorption and, therefore, an OC decrease, as given by:

2 $$OC=-1.26\times {10}^{-4}*{T}_{CA}+0.37.$$

Here, the values of $-1.26\pm 0.01 \times {10}^{-4} {K}^{-1}$ and $0.37\pm 0.01$ were related to the slope of Fig. 1b and the extrapolated value of OC at ideal ${T}_{CA}=O K$; respectively, these parameters were obtained from the fitting of the experimental data, as presented in Fig. 1a by employing Eq. (2). To verify the sensitivity of the results obtained in the adjustment of the OC characterization as a function of TCA, a second adjustment of these data was performed by the method of least squares, obtaining the same values of slope and intercept, with ${R}^{2}=1$. Figure 1S in complementary information.

The methodology hinges on an extrapolated oxide concentration (OC) value of 0.37 derived at an “ideal” synthesis temperature (${T}_{CA}=O K$). It’s important to acknowledge that extrapolations can introduce uncertainties, particularly when the available data doesn’t span a broad temperature range, as presented here in Fig. 1a. A comprehensive discussion is needed to establish the physical significance and reliability of this extrapolated value. Therefore, the value of 0.37 was derived mathematically by theoretical fitting employing Eq. (2), however, the physical interpretation of this value is related with a material that was not possible to generate, due to the FSTD method produce only polycrystalline GOF from RH at T_CA_ of 973 to $1273\, K$, and amorphous GOF from RH at T_CA_ of 773 to $923\, K$. Then, this value can be associated with the oxides and organic compounds that prevail on RH, as source material at ${T}_{CA}=0\,K$.

### Compositional and morphological analyses

Figure 1b presents the normalized XPS spectra of 11 GOF samples synthesized to T_CA_ from 773 to $1273 \,K$; peaks are observed at $\approx 538, 284\, eV$ associated with O1s and C1s respectively, evidencing the presence of carbon and oxygen atoms. Given that the general XPS spectra show the majority presence of carbon, as expected, high-resolution C1s and O1s spectra were performed for each of the 11 GOF samples.

Figure 1c and d shows the spectra corresponding to samples S-1173, with their respective deconvolution; the spectra were fitted by the Voigt function (GLP30). The C1s spectra show three bands in the BE range from 270 to 300 eV, these bands were associated with C sp^2^ ($284. eV$) and C sp^3^ ($285.2 \,eV$) hybridization and functional group of C─OH ($286.4 \,eV$)^33^. These values are within the ranges given by Johra, F. T. et al. in the reference^47^, taking into account the BE values characteristic of these functional groups presented and discussed by Stobinski in reference^33^. The O1s spectra generally show the presence of four bands in the range from 520 to $540 \,eV$; these bands were associated with functional groups of C═O ($531.9\pm 0.1 \,eV$), C─OH ($532.9\pm 0.2 \,eV$), C─O─C ($533.1\pm 0.4 \,eV$), and C─O ($534.2\pm 0.1 \,eV$), which supports the same interpretation and values reported by L. Stobinski et al. in reference^33^.

**Figure 2:**
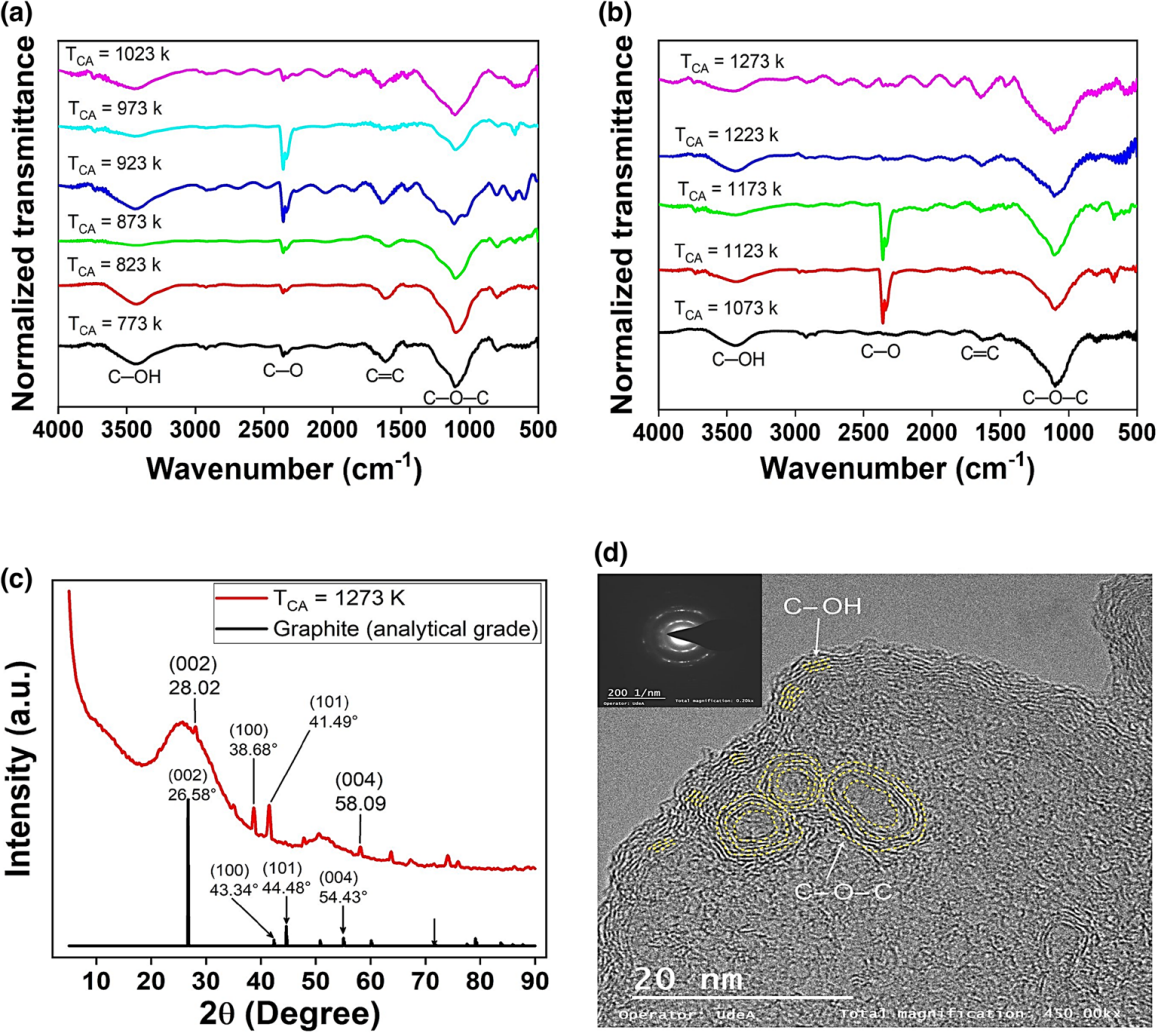
(**a**) and (**b**) FTIR spectra of GOF samples synthesized to T_CA_ from 773 to 1173 K, (**c**) XRD pattern of GOF samples synthesized at ${T}_{CA}=1273 \,K$ (in red) and graphite samples (in black), and (**d**) HR-TEM image in GOF sample synthesized at ${T}_{CA}=1123 \,K$ with 0.23 of OC showing formation of hydroxyl and epoxy bridges as parallel lines and curved patterns, respectively. Inset of figure (**d**), show electron diffraction image with characteristic concentric diffuse rings of polycrystalline structure in GOF sample.

The FTIR spectra displayed in Fig. 2a and b shows peaks localized at 3444, 2360, 1630 and $1099{ cm}^{-1}$, attributed to O─OH, C─O, C═C and C─O─C bonds respectively. The increase in T_CA_ is caused by the desorption of organic compounds and oxides, resulting in a decrease in C─OH (hydroxyl) bonds and an increase in C─O─C (epoxy) bonds. These results are consistent with the peaks reported before^48^.

Figure 2c shows a comparison between X-ray diffraction (XRD) patterns acquired in graphite analytical grade (in black) and GOF samples synthesized to ${T}_{CA}=1273 \,K$ (in red). It was found that GOF samples exhibit graphite structure, as expected. The GOF samples exhibit XRD peaks in the direction (002), (100), (101) and (004), similar to graphite-based materials^33,45^; the other peaks were associated with the oxide structural phase.

Figure 2d shows HR-TEM of the GOF sample at a scale of $20 \,nm$; finding, patterns of parallel and concentric ovals, associated to hydroxyl and epoxy bridges, respectively as reported previously^49^. Also, the inset of Fig. 2d, shows experimental results of electron diffraction in GOF samples, this experiment revealed concentric diffuse rings, associated to polycrystalline structure, as expected in graphene oxide materials^50^.

**Figure 3:**
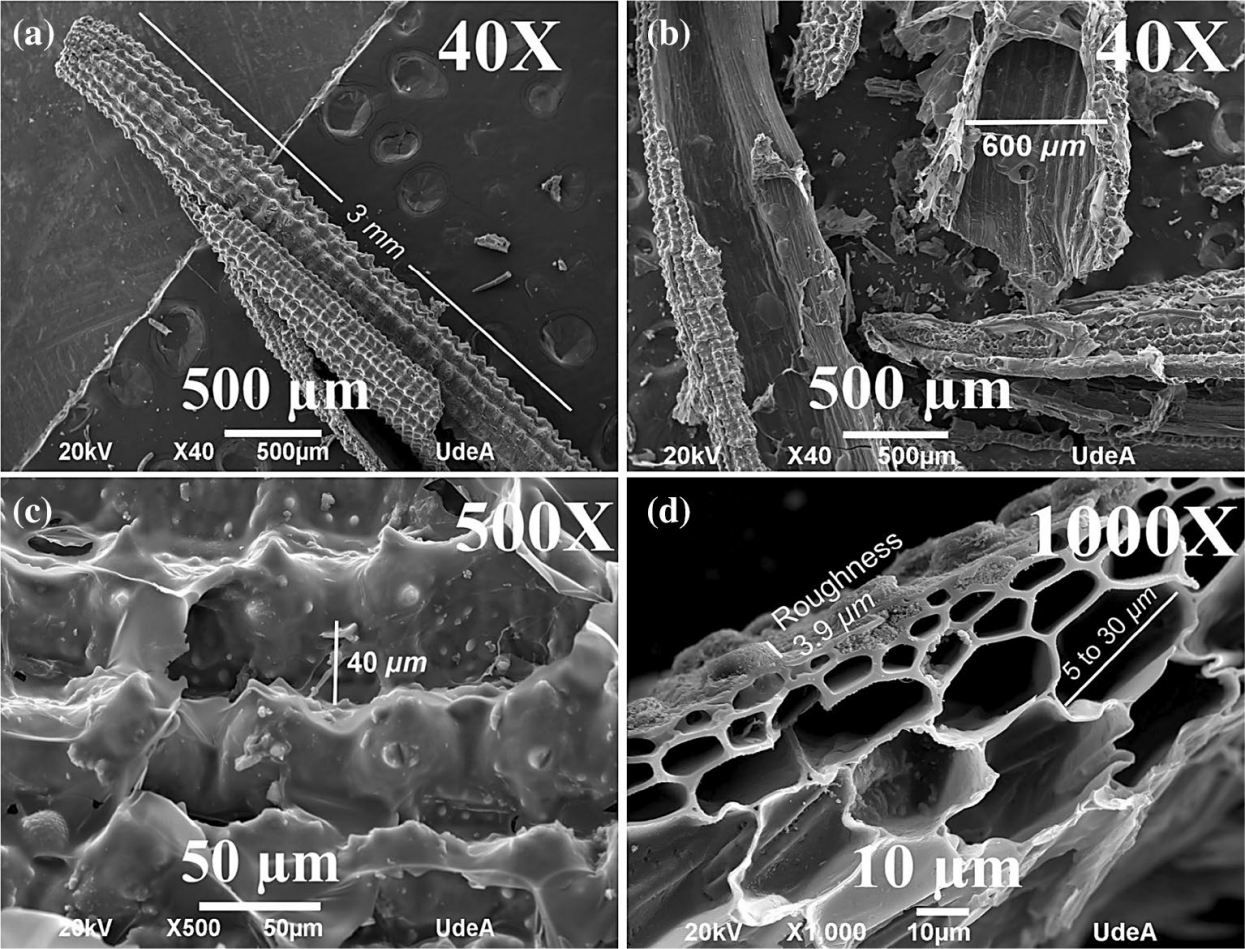
SEM micrographs of GOF samples S-973, the scale at (**a**) $500 \mu m 40X$, (**b**) $500 \mu m 40X$, (**c**) $50 \mu m 500X$, (**d**) $10 \mu m 1000X$.

Figure 3 presents the SEM micrograph of GOF samples S-973; it is possible to observe fibers with porous tubular and rough surface, lengths in the order of $3 \,mm$ and diameters of $600 \mu m$, surface corrugations with a size of $40 \mu m,$ approximately, as presented in Fig. 3a. The cross-section of the microchannels was observed with sizes varying from $1$ to $20 \mu m,$ as shown in Fig. 3b, and c shows the surface roughness. The microchannels exhibited porous structures with sizes from $5$ to $30 \mu m$, associated with transport pores, and roughness of $3.9\mu m$ as presented in Fig. 3d. However, the microporosity estimated by BET shows values of $2 \,nm$ as can be seen in supplementary information, Fig. S2. This characteristic morphology was observed in all GOF samples studied herein and is typical behavior of GO obtained from RH, as described by Ahiduzzaman M. et al. in reference^51^.

### Vibrational properties

**Figure 4:**
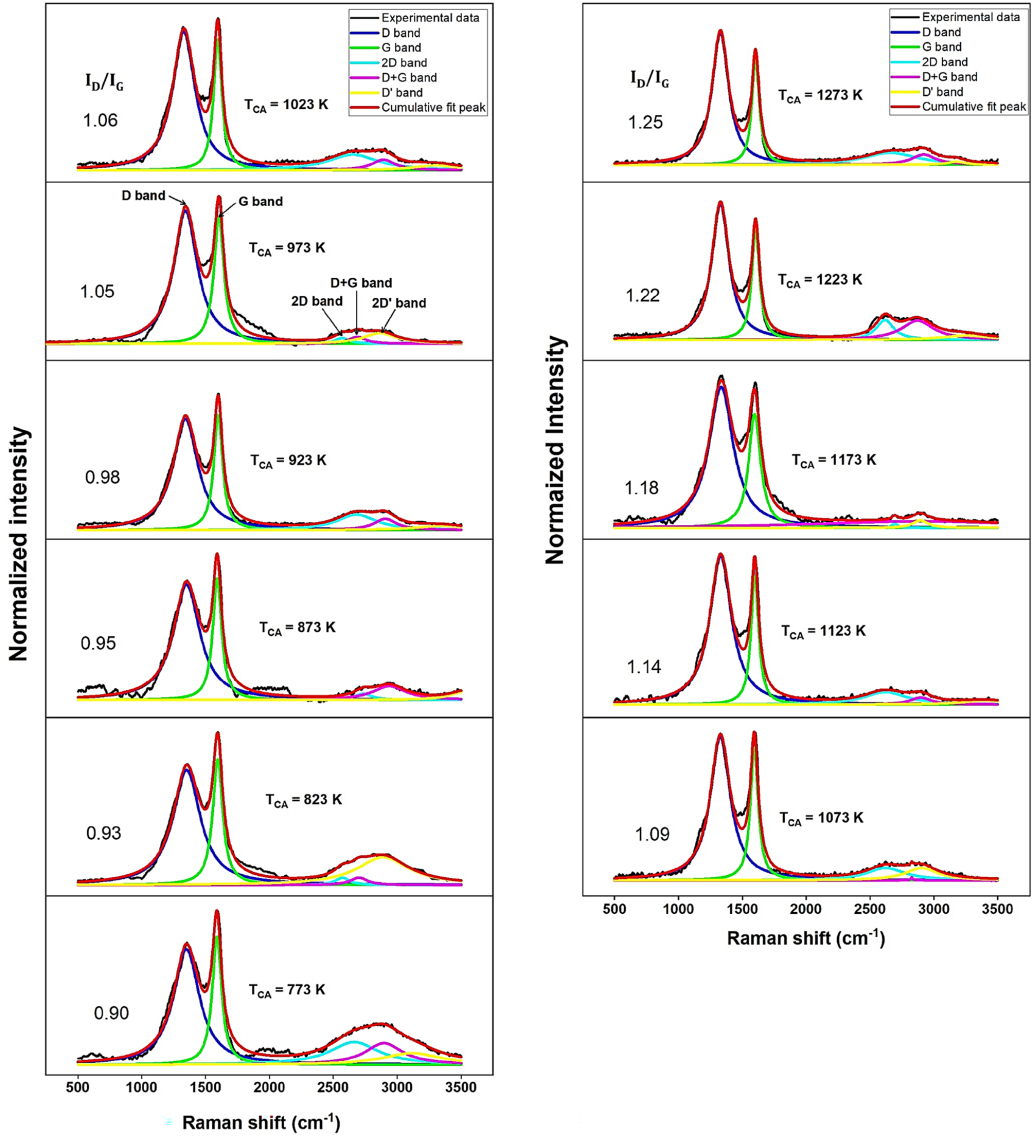
Raman spectra of GOF samples obtained by varying T_CA_ from 773 to 1273 K.

The Raman spectra of the GOF samples obtained by varying the T_CA_ from 773 to 1273 K are shown in Fig. 4. The fitting and deconvolution of the Raman spectra served to identify the main vibrational contributions. The d-band ranged from $1329$ to $1350\, {cm}^{-1}$ for samples with lowest OC (S-1273) and highest OC (S-773), respectively. The G-band varied from $1600$ to $1589\, {cm}^{-1}$, for lowest OC and highest OC, respectively, as reported by T. Liou and P. Wang in^8^. The state of the D-band is caused by the presence of defects (disorders, vacancies, and functional groups) and, according to M.S. Ismail et al. in^29^, its height depends on the number of the sp^3^ carbon atoms of graphene surface and the number of the defects of the graphene^36^ while the G-band is caused by the formation of the stretching vibration sp^2^ carbon atoms and represents the graphitized carbon^36^. These bands are characteristic of the Raman spectrum of GO, corresponding to the symmetry A1g and the vibrational mode of E2g, respectively, and overtone bands at high Raman shift of $2622\, {cm}^{-1}$ (2D band), $2875\, {cm}^{-1}$ (D + G band), and $3100\, {cm}^{-1}$ (2D’ band), according to data reported in^23^. Broadening of these bands is related with the stacking effect of GO monolayers with edges, defects, and sp^2^ regions^23,52^.

D-band broadening from $FWHM=156.45\, {cm}^{-1}$ at the lowest OC (0.21, at ${T}_{CA}=1273\, K$) to $FWHM=245.35\, {cm}^{-1}$ with ${R}^{2}=0.98863$, at the highest OC (0. 28, at ${T}_{CA}=773 K$) with ${R}^{2}=0.98217$. This behavior can be attributed to the increment of the dispersive process by incrementing the OC. Since this is a light scattering process, we find that Lorentz-type functional forms describe these experimental Raman spectrum data very well.

### Electrical properties

**Figure 5:**
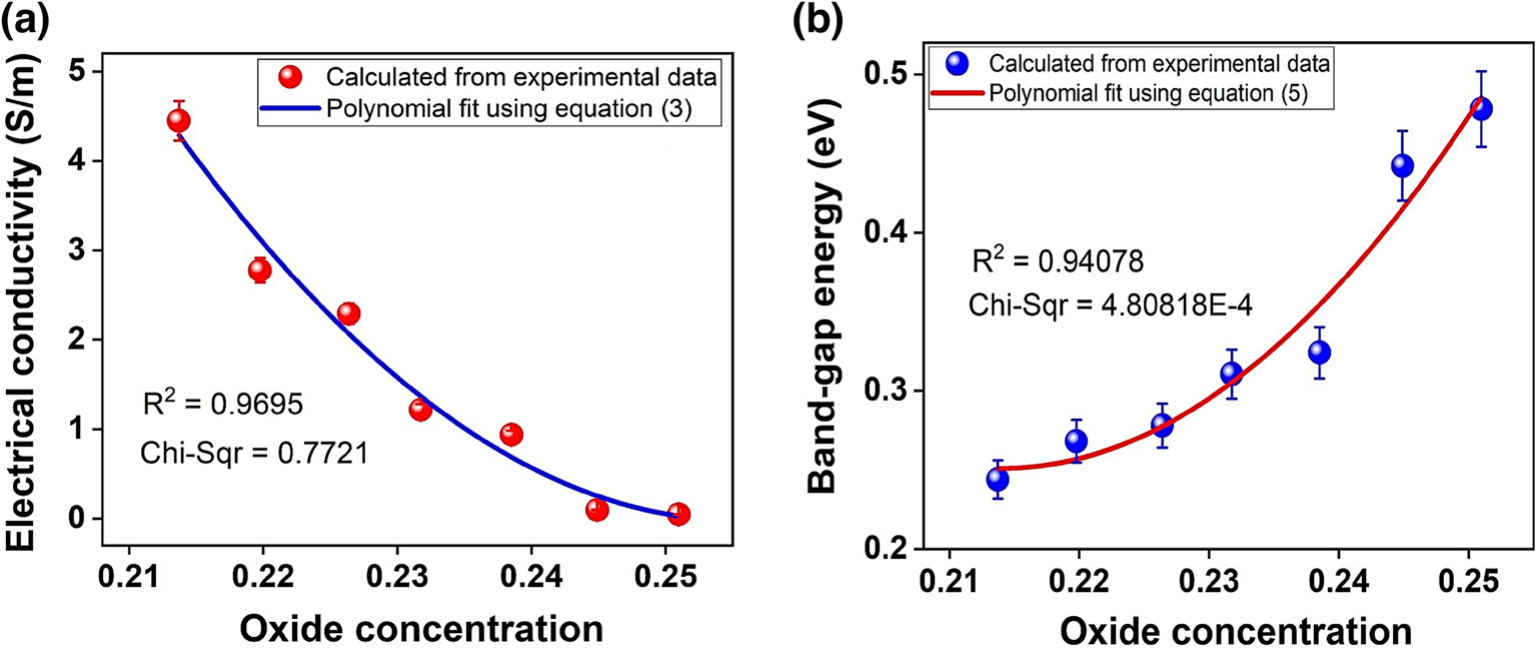
Influence of OC on: (**a**) Electrical Conductivity and (**b**) Band-gap Energy of GOF samples.

Figure 5a shows the electrical conductivity variation of GOF samples as a function of the OC. It was found that it decreases OC from 0.25 to 0.21 and increases electrical conductivity from $4.66\times {10}^{-2}$ to $4.45 S {m}^{-1}$. These correspond to a change of two orders of magnitude. Since the use of GOF materials for the development of advanced electronics of sensors and devices requires low electrical resistance and high electrical conductivity, the correlation between the OC and the electrical conductivity is shown in Fig. 5a; this corresponds to a change non lineal behavior of two orders of magnitude in response to a variation in OC from 0.25 to 0.21. Therefore, the best OC value for GOF samples is 0.21, given that it exhibits the maximum of electrical conductivity at 4.45 S m^–1^. This behavior, $\sigma =f\left(OC\right)$, is similar to that reported for graphene oxide obtained through other synthesis methods^35,39,48,49^. This increase in electrical conductivity can be attributed to the desorption of oxides and organic compounds via thermal decomposition, as a consequence of T_CA_ variation, which modifies the OC, as reported^53^. It is well known that decreasing the OC increases the interatomic distances between the carbon atoms, which increases the mean free path and the relaxation time of the charge carriers into boundary defects, which increases the electrical conductivity. In addition, the reduction in OC decreases the crystallite size ($4.20$ to $3.52 \,nm$), which increases the density of boundary defects, these boundary defects appear in our materials at the atomic scale, as a consequence of the presence of hydroxyl and epoxy bridges, which form patterns of straight parallel lines and concentric circles; respectively, as proposed by Hoyos-Ariza, et al.^49^.

The experimental data of GOF electrical conductivity was fitted employing a polynomial function, as presented by the blue curve in Fig. 5a, as expected and to describe the $\sigma =f\left(OC\right)$ relation in a semiconductor material, as reported by Van Vechten^54^. Thus, it was found that electrical conductivity as a function of OC in GOF samples can be described by Eq. (3).

3 $$\sigma \left(x\right)=d+fx+g{x}^{2}.$$

Here, $x$ is the independent variable associated with OC; $d$ corresponds to electrical conductivity independent of the OC, with a value of $161.9\pm 0.1 S/m$; $f$ was related to the linear factor of the OC that corresponds to a value of $-1268.4\pm 0.1S/m$; and $g$ is associated to the nonlinear factor of the OC with a value of $2483.5\pm 0.1 S/m$. The best fit was obtained with ${R}^{2}=0.9695$, as proposed here.

Equation (4) was used to calculate the ${E}_{g}$ of the GOF samples, described by^21^.

4 $$\sigma ={\sigma }_{0}{K}_{B}T*exp\left(\frac{-{E}_{g}}{2{K}_{B}T}\right).$$

where ${E}_{g}$ is the band-gap energy, $\sigma$ is the electrical conductivity of the GOF samples presented in Fig. 5a, ${\sigma }_{0}$ is the electronic conductivity independent of temperature, ${K}_{B}$ is the Boltzmann constant, and $T$ is the temperature.

As seen in Fig. 5b, the ${E}_{g}$ of the GOF samples varies as a function of OC. Band-gap energy shows a variation from $0.24 \,eV$ to $0.48 \,eV$ by increasing the OC from 0.21 to 0.25, presenting similar behavior to that reported by theoretical studies, which have predicted that increased ${E}_{g}$ is related to increased oxidation, as reported in references^4,48,49,55^. Also, as reported before^48^ the UV–vis measurement in graphene oxide materials revealed responses in the range of 1.3 to 4.4 eV^56^, which correspond to energy transitions in other critical points (i.e. E1 or E1 + ΔE1)^48^; however, it is necessary to perform more experiments with other optical characterization techniques, to confirm the nature of these results, this aspect will be published in a future work. The experimental data of ${E}_{g}\left(x\right)$ was fitted by employing a quadratic function. As illustrated by the red curve of Fig. 5b, it was found that the Van Vechten model describes the experimental results effectively, and it revealed that GOF samples exhibit semiconductor behavior, as expected and given by^54^.

5 $${E}_{g}\left(x\right)=a+bx+c{x}^{2}.$$

Here, $x$ is the independent variable associated with OC; $a$ corresponds to the ${E}_{g}$ independent of the OC with a value of $8.1\pm 0.1 \,eV$; $b$ was related with the linear factor of the OC that corresponds to a value of $-73.1\pm 0.1 \,eV$; and $c$ is associated with the nonlinear factor of OC with a value of $170.8\pm 0.1 \,eV$. The best fit was obtained with ${R}^{2}=0.94078$. It is evident that OC modifies the electrical properties of GOFs, as expected^57,58^.

**Figure 6:**
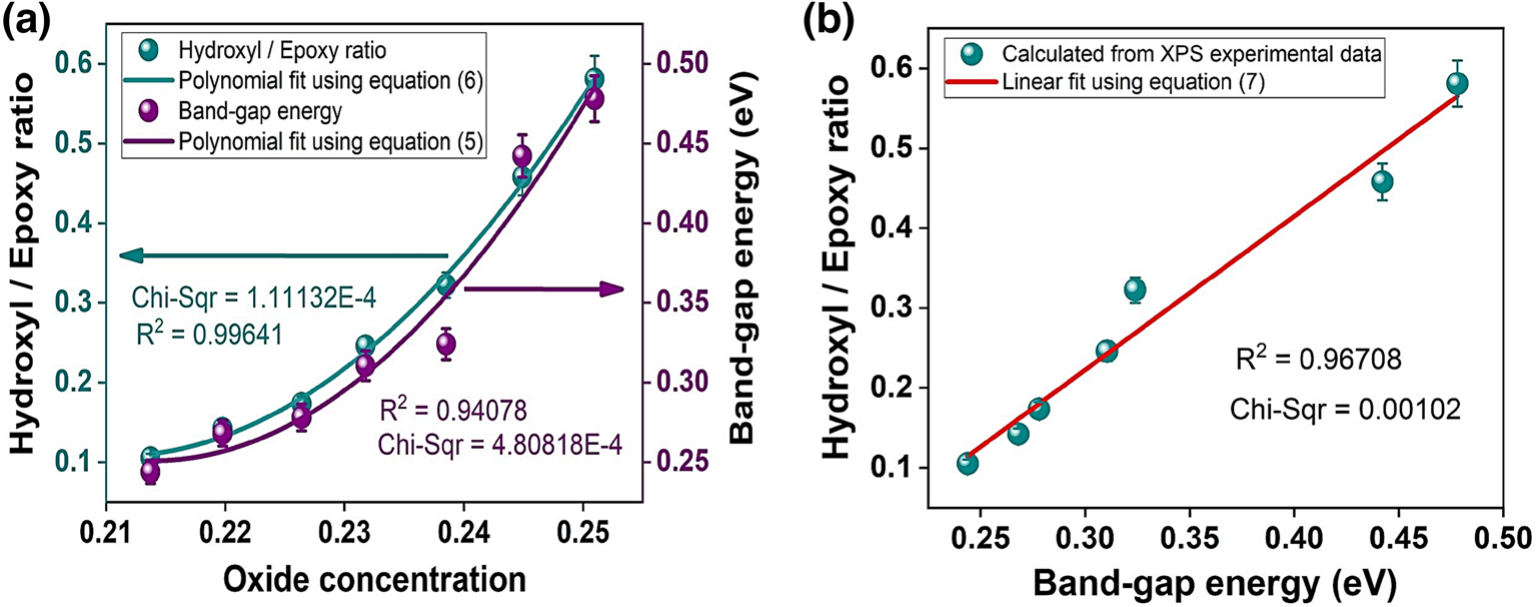
(**a**) Influence of OC on ${E}_{g}$ and hydroxyl/epoxy ratio, (**b**) Correlation between ${E}_{g}$ and hydroxyl/epoxy ratio in GOF samples.

In order to elucidate the possible mechanism responsible for semiconductor behavior in GOF samples, measured at room temperature, a correlation was conducted among the influence of OC with ${E}_{g}$ and hydroxyl-epoxy ratio, as presented in Fig. 6a. Previous studies reported that oxides in graphene are mainly hydroxyl and epoxy, as functional groups^59^; the presence of these oxides increase the interplanar distance^60^. The hydroxyl/epoxy ratio was estimated by employing the area comparison method of XPS spectra as $\left[1-\left(\frac{{A}_{-O-}}{{A}_{OH}}\right)\right]$ for each GOF sample, as reported^61^. Figure 6a. shows an ${E}_{g}\left(x\right)$ scale with hydroxyl/epoxy ratio, as a function of OC. Consequently, we believe that the presence of multifunctional oxides increases the ${E}_{g}$, as expected for a semiconductor material^48^, and these changes in the electrical properties of GOF samples can be attributed to the formation at atomic scale of hydroxyl bridges promoted by experimental T_CA_. The presence of hydroxyl bridges in GOF samples at an atomic scale rearranges the graphene structure and out-of-plane carbon atoms, reducing the average interatomic distance opening the ${E}_{g}$, as reported on graphene oxide obtained from bamboo^61^. The experimental data of the hydroxyl/epoxy $\left(\frac{H}{E}\right)$ ratio was fitted by employing a polynomial function, as described by the green line in Fig. 6a, given by the equation:

6 $$\left(\frac{H}{E}\right)\left(x\right)=m+nx+p{x}^{2}.$$

Here, $x$ is the independent variable associated with OC; $m$ corresponds to the hydroxyl/epoxy ratio independent of the OC with a value of $12.9\pm 0.1$; $n$ was related with the linear factor of the OC that corresponds to a value of $-121.9\pm 0.1$; and $p$ is associated with a nonlinear factor of the OC with a value of $289.5 \pm 0.1$. The best fit was obtained with ${R}^{2}=0.99641$, as proposed here.

The correlation between $\left({E}_{g}\right)$ and hydroxyl/epoxy ratio, $\left(\frac{H}{E}\right)$, shows that decreased ${E}_{g}$ from $0.48 eV$ to $0.24 eV$, increases $\frac{H}{E}$ from $0.11$ to $0.58$, Fig. 6b. The experimental data of $\frac{H}{E}$ was fitted by using a linear function, as described by the red line in Fig. 6b, given by the equation:

7 $$\left(\frac{H}{E}\right)\left(x\right)=q+rx.$$

Here, $x$ is the independent variable associated with ${E}_{g}$; $q$ corresponds to the $\frac{H}{E}$ independent of the ${E}_{g}$ with a value of $-0.36\pm 0.01$; $r$ was related with the linear factor of the $\frac{H}{E}$ that corresponds to a value of $1.92\pm 0.01$. The best fit was obtained with ${R}^{2}=0.96708$, as proposed here.

### Correlation among oxide concentrations, vibrational, and electrical properties

The Raman crystal size and boundary defect density were calculated by employing the Eqs. (8) and (9), respectively, for each GOF sample at different T_CA_, given by^21^.

8 $${L}_{A}\left(nm\right)=4.4\left(\frac{{I}_{G}}{{I}_{D}}\right),$$

9 $${{n}_{D}}^{2}\left({cm}^{-2}\right)=107.57\times {10}^{-9}\left(\frac{{I}_{D}}{{I}_{G}}\right).$$

**Figure 7:**
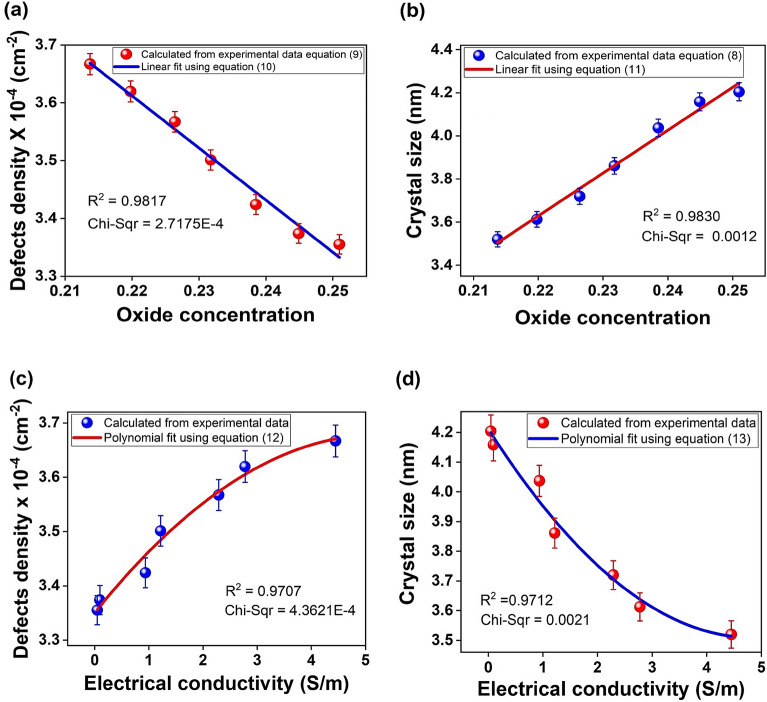
Correlation among OC and (**a**) Defects density and (**b**) Crystal size; and correlation among electrical conductivity and (**c**) Defects density and (**d**) Crystal size of GOF samples.

Here, ${I}_{D}$ is the normalized intensity of the D-band and ${I}_{G}$ is the normalized intensity of the G-band. The increased $\frac{{I}_{D}}{{I}_{G}}$ ratio in Eqs. (8) and (9) represents the transformation of a disordered structure into an ordered one, as expected. The correlation between OC and vibrational properties shows that a decreased OC increases the boundary defects density from $3.35$ to $3.67\times {10}^{-4} {cm}^{-2}$, as presented in Fig. 7a, and decreases crystal size from $4.2$ to $3.52 nm$, as shown in Fig. 7b. This can possibly be explained by the desorption of multifunctional oxides and some organic compounds due to thermal decomposition methods employed to synthesize GOF samples, as expected^60^. The experimental defects density data were fitted with a linear function, described by the blue line in Fig. 7a, given by Eq. (10).

10 $${n}_{D}\left({cm}^{-2}\right)\left(x\right)=\alpha +\gamma x.$$

where $x$ is the independent variable associated with OC; $\alpha$ corresponds to the independent term of the OC with a value of $5.59\pm 0.01 {cm}^{-2}$; $\gamma$ was related with the linear factor of the OC that corresponds to a value of $-9.01\pm 0.01 {cm}^{-2}$. The best fit was obtained with ${R}^{2}=0.9817$, as proposed here. The experimental crystal size data were fitted with a linear function described by the red line in Fig. 7b and given by Eq. (11).

11 $${L}_{A}\left(nm\right)\left(x\right)=\delta +\varepsilon x.$$

Here, $x$ is the independent variable associated with OC; $\delta$ corresponds to the independent parameter of the OC with a value of $-0.74\pm 0.1 nm$; $\varepsilon$ was related with the linear factor of the OC that corresponds to a value of $19.88\pm 0.1 nm$. The best fit was obtained with ${R}^{2}=0.9830$, as proposed here. The correlation between oxide concentration and vibrational properties can be described by expressions (10) and (11), as proposed here.

The correlation between vibrational properties and electrical conductivity, shows an increase in boundary defects density from $3.35$ to $3.66{\times 10}^{-4} {cm}^{-2}$ as shown in Fig. 7c and decreases in crystal size from $4.20$ to $3.52 nm$, as presented in Fig. 7d, due to the decrease in the OC; which leads to an increase in electrical conductivity from $4.66\times {10}^{-2}$ to $4.45 \frac{S}{m}$, as presented in Fig. 7c and d. This behavior can be attributed to the fact that low oxidation increases the interatomic separation between carbon atoms, which improves the characteristic relaxation time and electrical conductivity. The increased separation of carbon atoms also reduces the crystallite size (4.20 to $3.52 nm$), and the density of boundary defects is increased by low oxidation, while high OC decreases this characteristic time, due to a higher presence of impurities, mainly hydroxyl groups. These behaviors are very important, demonstrating that electrical and vibrational properties are tuned by OC variation; also, revealing the multifunctional effect of hydroxyl and epoxy groups present in GOF samples.

The experimental defects density data were fitted with a polynomial function described by the red line in Fig. 7c and given by Eq. (12).

12 $${n}_{D}\left({cm}^{-2}\right)\left(\sigma \right)={n}_{D}\left(0\right)+\varphi \sigma +\nu {\sigma }^{2}.$$

where $\sigma$ is the independent variable associated with electrical conductivity; ${n}_{D}\left(0\right)$ corresponds to the independent term of the electrical conductivity with a value of $3.35\pm 0.01 {cm}^{-2}$; $\varphi$ was related with the linear factor of the electrical conductivity, that corresponds to a value of $0.12\pm 0.01\frac{ m}{S}{cm}^{-2}$; and $\nu$ is associated with the nonlinear factor of the electrical conductivity with a value of $-0.01\pm 0.01 \frac{{m}^{2}}{{S}^{2}}{cm}^{-2}$. The best fit was obtained with ${R}^{2}=0.9707$, as proposed here.

The experimental data the crystal size was fitted by employing a polynomial function, as described by the blue line in Fig. 7d and given by:

13 $${L}_{A}\left(nm\right)\left(\sigma \right)={L}_{A}\left(0\right)+l\sigma +\kappa {\sigma }^{2}.$$

Here, $\sigma$ is the independent variable associated with electrical conductivity; ${L}_{A}\left(0\right)$ corresponds to the independent term of the electrical conductivity with a value of $4.21\pm 0.01 nm$; $l$ was related with the linear factor of the electrical conductivity that corresponds to a value of $-0.29\pm 0.01 \frac{m}{S}nm$; and $\kappa$ is associated with the nonlinear factor of the electrical conductivity with a value of $0.03\pm 0.01 \frac{{m}^{2}}{{S}^{2}}\mathrm{nm}$. The best fit was obtained with ${R}^{2}=0.9712$, as proposed here. The correlation between electrical conductivity and vibrational properties can be described by expressions (12) and (13), as proposed here.

### Heater device based on GOF

**Figure 8:**
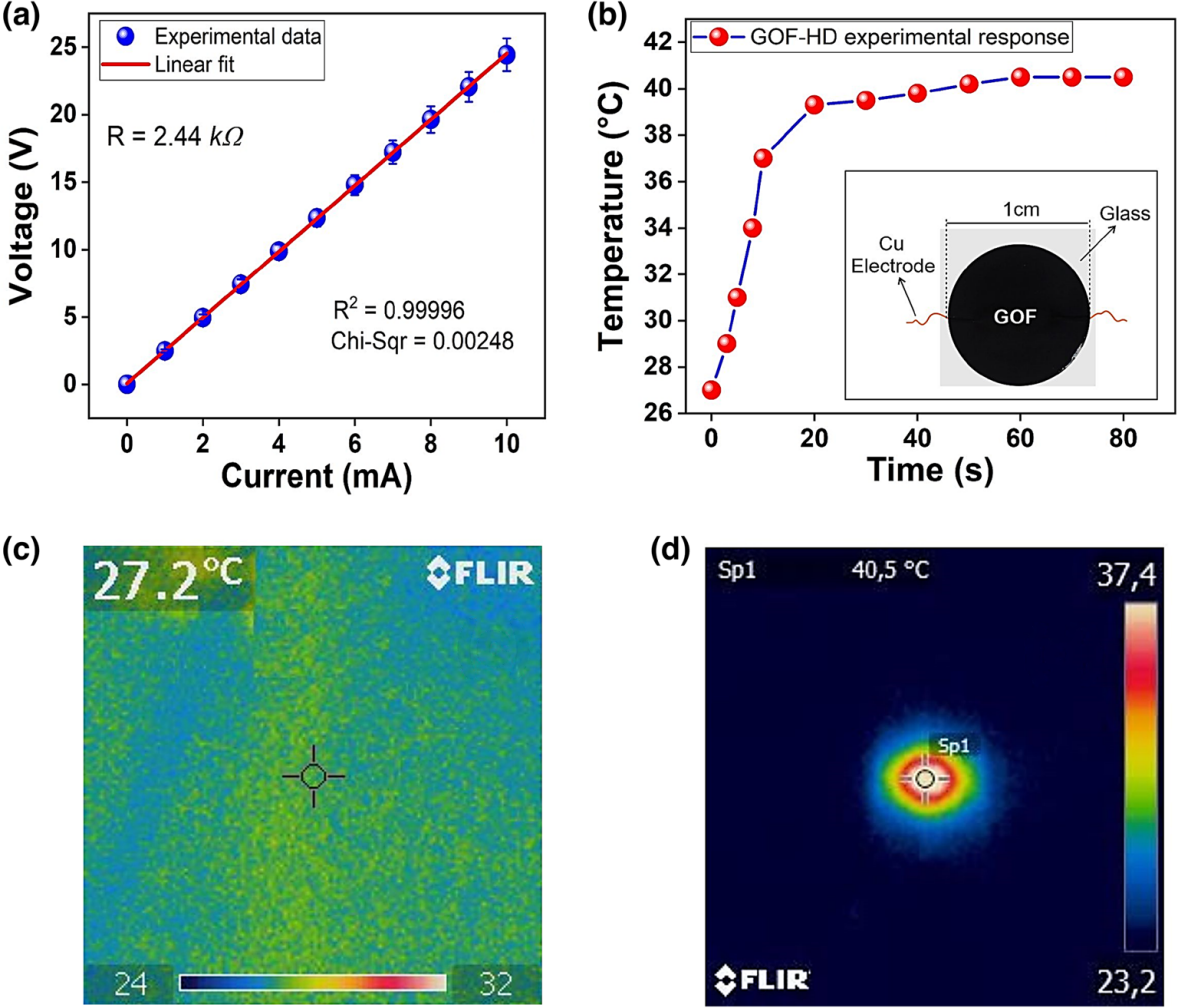
(**a**) Electrical characterization. (**b**) Temperature evolution in GOF-HD with external applied direct current at $10 mA$ and voltage of $2.44 V$. The inset show the digital photographic of GOF-HD. (**c**) Thermographic imagen GOF-HD power off. (**d**) Thermographic imagen power GOF-HD on.

The electrical response of the GOF-based electrically controlled ohmic device^62^ based in GOF (GOF-HD) shows a variation of the voltage between its terminals in response to the variation of the applied current flowing through it. The well-known Ohm's law states that the voltage across an electrical resistance is directly proportional to the current flowing through it $\left(V=iR\right)$, and that the constant of proportionality corresponds to the value of the electrical resistance of the device. GOF-DH has a linear response, Fig. 8a, according to Ohm's law, with an intercept at the origin and a positive slope, i.e., the voltage between the terminals of the device increases proportionally to the increase in the electric current applied, going from $0 V$ when the current is zero $\left(i=0\right)$ to $24.44 V$ when the current is of $10 mA$. The sensitivity of GOF-HD, corresponding to the slope of its linear response, is of $2.44 k\Omega$, as expected^4^.

Figure 8b presents the thermal behavior of the GOF-HD when a direct current of $10 mA$ is applied between its terminals, which generates a thermal variation from room temperature, 27.2 to 40.5 °C, in a time of 80 s. The temperature evolution can be associated with the behavior of a first-order system, characterized by a transient between 0 and 20 s, and a stationary state with a response around 40.5 after 20 s; i.e. the temperature variation in the device is significant in the transient state and minimal in the stationary state. These results agree with those previously reported^4^. The inset of Fig. 8b shows the digital photographic of GOF-HD proposed here, with electrical contacts. Figure 8c and d present the thermographic images when the GOF-HD is power-off and power-on, which exhibits temperatures of $27.2^\circ{\rm C}$ and $40.5^\circ{\rm C}$ at stationary response; respectively. The heater behavior can be explained by the Joule effect, as reported before^4^, which is related to the heat produced in the IR emitting device, as a consequence of the circulation of an electric current through it. The GOF-HD exhibits a higher temperature variation $\left(15.3^\circ{\rm C} \right)$ than that of the IR emitter based on graphene oxide synthesized from bamboo pyroligneous acid reported in reference^4^, when a current of 10 mA is applied to it.

Based on the results obtained in the GOF-HD presented here, it is possible to suggest potential applications such as the development of heater flexible devices using diverse geometries and deposition method^63^, and industrial applications in gas sensing^62,63^, thermal therapy, biological, electrical and mechanical sectors, food industry, and water treatment^62^, among others.

## Materials and methods

The GOF samples were synthesized via the FSTD method in an automated pyrolysis system with a controlled nitrogen atmosphere. As a result of several tests and analyses, a protocol for the synthesis of GOF samples from RH was established. Once the RH is deposited in the reactor, a vacuum is created in the system, then nitrogen is allowed to flow through the system with the vacuum system operating, and finally the vacuum action is released and the nitrogen flow is continued until the inert atmosphere is achieved. The execution of this protocol generates an inert atmosphere to avoid the thermal decomposition of the material and achieve its carbonization. Since RH contains organic matter and 20% inorganic matter, a carbonization yield between 39.3 and 26.3% was obtained by varying the T_CA_ from 773 to $1273 K$. Reportedly, the main elemental components of RH are C $37.05 wt.\%$ and O $35.03 wt.\%$, N $11.06 wt.\%$, Si $9.01 wt.\%$, and H $8.80 wt.\%$; which makes it a powerful material in the production of carbon-based materials^64^. Research has been reported on the use of RH to obtain activated carbons with significant surface areas and interesting properties, which have been used in the development of supercapacitors^5,65,66^, electrodes for electric batteries^18^, and gas sensors^43^, among others. In addition, preliminary studies have shown that it is possible to obtain graphene-based materials from biomass elements using the thermal decomposition method, which is recognized in the reported scientific literature^28,48,67–69^.

The GOF samples were mechanically ground in a mortar to a particle size $<180 \mu m$, which was verified by passing the material through a Ro-Tap Model E sieve. In all the procedures and methods applied in the development of this work, the relevant standards were considered. The GOF samples were coded as S-T_CA_, for example, code S-973 corresponds to a sample synthesized to 973 K. The authors confirm that all methods in experimental research and field studies on waste products of the commercial rice husk were performed in accordance with the relevant regulations.

### Characterization techniques

To obtain the SEM micrographs, the samples were fixed onto a graphite tape, a thin gold (Au) coating was applied (DENTON VACUUM Desk IV equipment) and they were analyzed in the JEOL JSM 6490 LV scanning electron microscope, using a high vacuum to obtain the images. The secondary electron detector was used to evaluate the morphology and topography of the samples.

The compositional analysis was performed through XPS, using a Spec photoelectron X-ray spectrometer (NAP-XPS) with a monochromatic Al Kα $hv=1486.7 \,eV$ source. Raman measurements were carried out at room temperature by employing a confocal Horiba Jobin Yvon, Model Labram HR, Raman spectrometer with an excitation HeNe laser beam working at a $632 \,nm$ wavelength and $17 \,mW$. All spectra were acquired under the same conditions in a range from 500 to 3500 ${cm}^{-1}$. The sheet resistance value was measured by electrical characterization using the four-point method with collinear electrical contacts. In this method, a constant current is applied to two of the tips, and the potential of the other two tips is measured with a high-impedance voltmeter. The powered GOF samples were compacted to $1 \,kg$ force. Measurements were performed by injecting electrical current in the range of $-100$ to $+100 nA$ at four points as an electrical contact configuration^21^. For X-ray diffraction (XRD) analysis, Co Kα radiation (λ = 1.78901 Å) was used between 10° and 80° 2θ, with steps of 0.0260° and a step time of $41.5660 s$, with a current of $40 \,mA$ and a voltage of $45 \,kV$ 45. For the XPS, Raman, XRD, and electrical conductivity analyses, the GOF samples were mechanically ground in a mortar to a particle size of < 180 µm, verified by passing the material through a Ro-Tap Model E sieve. The FTIR measurements were performed by using an FTIR is50 FT-IR Nicolet. Thermo Scientific, equipment, detection range $4000-400{cm}^{-1}$, optical velocity $0.474 \frac{cm}{s}$, a tablet was prepared to obtain the IR spectra of each GOF sample by preparing a 1:5 mixture of KBr standard. The HR-TEM analysis. Transmission electron microscopy (TEM) and high resolution (HR)-TEM images were obtained using a Tecnai F20 Super Twin TMP on an FEI microscope, field emission source, $0.1\,nm$ resolution at $200 \,kV$, maximum TEM magnification 1.0 MX, GATAN US 1000XP-P camera. Oxford Instruments XMAX EDX detector. STEM analysis—FISCHIONE Instruments Model M3000 FP5360/22 HAADF detector 120/200 kV. The GOF samples were sonicated for 10 min, then a portion of the solution was removed and an aliquot was placed on the 200-mesh copper lacey carbon grid and allowed to dry before being mounted in the instrument.

### Heater device configuration

**Figure 9:**
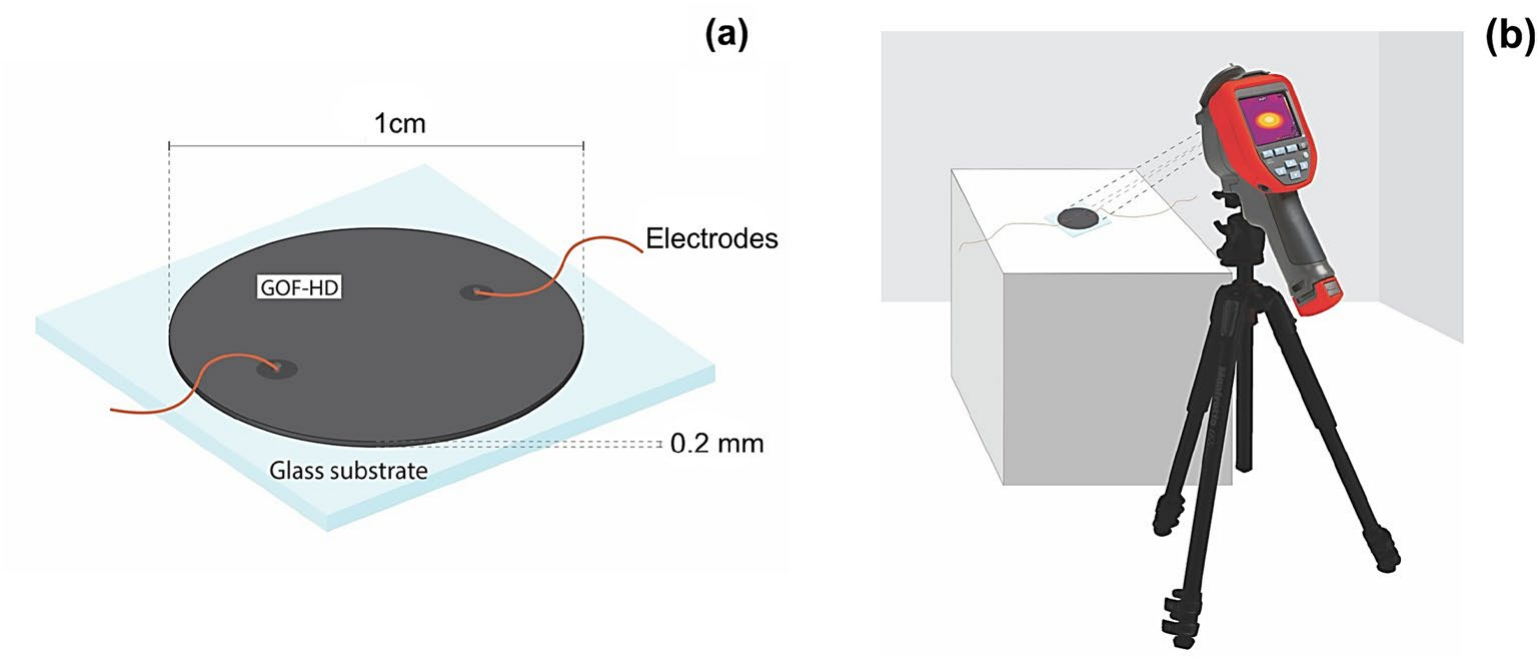
(**a**) Schematic diagram of the configuration of the GOF-HD developed here. (**b**) Experimental setup used for controlled thermographic image acquisition.

The GOF-HD^62^ was developed here employing an ink based on GOF samples, which was obtained by mixing 0.5 g of GOF samples, $0.7 \,ml$ of acetone and $0.5 \,ml$ of nail polish as a binder. This ink was deposited by using a painting method on a glass substrate to form a circular structure with $1 \,cm$ of diameter and $0.2 \,mm$ of thickness, the electrical contact was carried out by two wire copper electrodes into ink, interconnected as shown in Fig. 9a. The electrical measurement was carried out via the two-point I-V curves method, by two-probe configuration in a range from 0 to $10 mA$, using current sources and nano-voltmeter Keythley Instruments Inc, 6221 and 2182A respectively. The thermal response was measured with infrared thermography FLIR-2007 with a controlled image acquisition in two states, power off and power on ($10 mA$ and $24.44 V$, fixed), Fig. 9b shows a diagram of the experimental setup used here.

## Conclusions

Graphene oxide fiber samples were synthesized from RH biomass, employing the first-stage thermal decomposition method. The experimental carbonization temperature varies the OC of GOF samples, and a linear relationship was found between these two synthesis parameters. The XPS spectra of GOF samples show two main peaks corresponding to C1s and O1s. It was found that decreased carbonization temperature increases oxygen content; the deconvolution of XPS spectra shows peaks for C1s corresponding to C sp^2^, C sp^3^, and C–OH, while for O1s, the main peaks are C–OH, C–O–C, and C–O. The GOF samples exhibit a morphology of fibers composed of porous structures with sizes between $5$ and $30 \mu m$, the vibrational response of graphite oxide materials, with crystal size from $3.52$ to $4.88 \,nm$ and the boundary defects density of $3.12-3.67\times {10}^{-4} {cm}^{-2}$. When OC decreases from 0.25 to 0.21, it increases electrical conductivity from $4.66\times {10}^{-2}$ to $4.45 \,S/m$, decreases the ${E}_{g}$ from $0.48 \,eV$ to $0.24 \,eV$, increases the defects density from $3.19\times {10}^{-4}$ to $3.58\times {10}^{-4} {cm}^{-2}$, and decreases crystal size from $4.88$ to $3.52 \,nm$; these behaviors are possibly attributed to the desorption of oxides and some organic compounds, as well as to the presence of hydroxyl and epoxy groups, by the oxidation processes. The correlation between ${E}_{g}$ and hydroxyl/epoxy ratio suggests a semiconductor behavior of GOF samples, as expected and attributed to the presence of the hydroxyl bridges at atomic scale, which were tuned by experimental T_CA_. Likewise, the correlation between electrical conductivity and vibrational properties, such as defects density and crystal size, revealed that increased electrical conductivity increases defects density and decreases crystal size; this behavior may be attributed to the increased characteristic relaxation time of the dispersive processes. This increases electrical conductivity by reducing OC, which reduces crystal size and, thus, increases the boundary defects density in GOF samples. Consequently, the OC significantly affects the electronic structure and vibrational properties of GOF samples. By tuning OC, it was possible to control the electrical conductivity and the ${E}_{g}$. These results suggest that GOF samples are a possible candidate material for the development of advanced electronics of sensors and devices, such as heater devices or IR emitter.

### Supplementary Information

Supplementary Information.

## Data Availability

The datasets used and/or analyzed during the current study available from the corresponding author on reasonable request.
