# Supplementary material for: Basic physical properties and potential application of graphene oxide fibers synthesized from rice husk
Source: Sci Rep. 2023 Oct 20;13:17967. doi: 10.1038/s41598-023-45251-8 (PMC10589357; doi:10.1038/s41598-023-45251-8)
Supplement: Supplementary file 1 — Supplementary Information. [file 41598_2023_45251_MOESM1_ESM.docx]

Basic physical properties and potential application of graphene oxide fibers synthesized from rice husk

**Suplementary information**


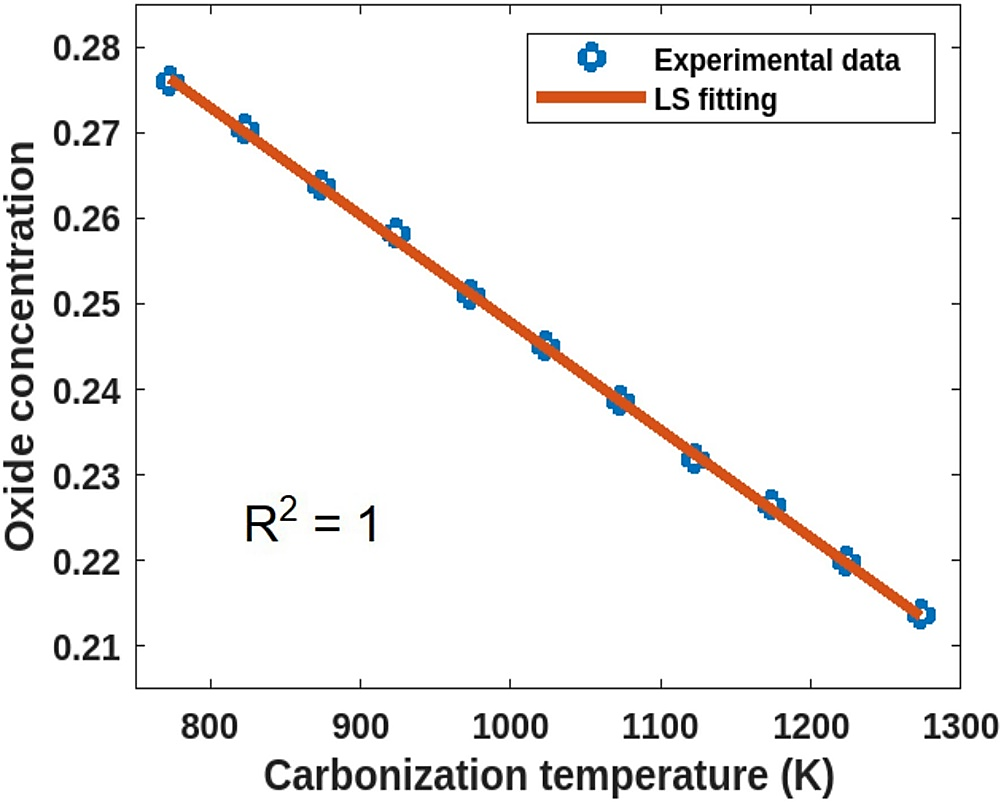


**Fig. 1S.** Carbonization temperature influence in OC of GOF samples obtained by varying T_CA_ from 773 to 1273 K, fitted by the least square’s method.

|  | $OC=-1.26\times{10}^{-4}*T_{CA}+0.37.$ |  |
| --- | --- | --- |


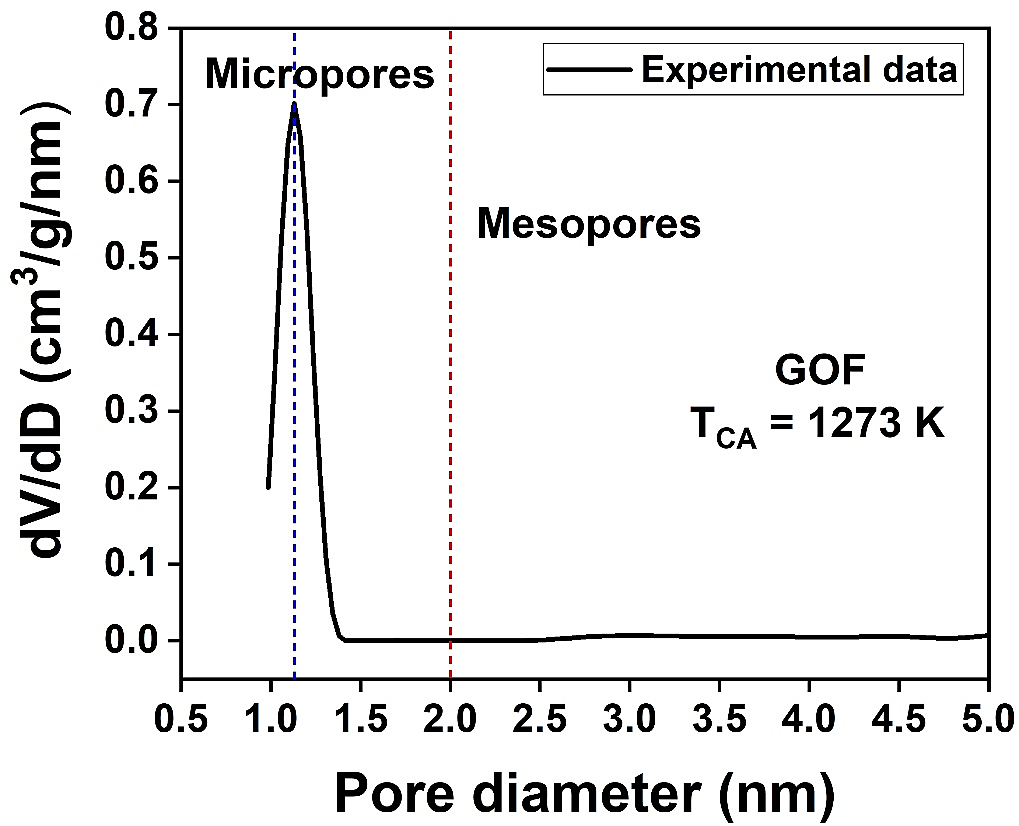


**Fig. 2S.** Pore size distribution, GOF sample synthesized at $T_{CA}=1273 K$
